# The Effect of Coronavirus 2019 Disease Control Measures on the Incidence of Respiratory Infectious Disease and Air Pollutant Concentrations in the Yangtze River Delta Region, China

**DOI:** 10.3390/ijerph19031286

**Published:** 2022-01-24

**Authors:** Lan Wang, Kehan Wang, Hui Zhong, Na Zhao, Wangli Xu, Yunmei Yang, Yiran He, Shelan Liu

**Affiliations:** 1 Department of Geriatrics, First Affiliated Hospital, Zhejiang University School of Medicine, Hangzhou 310003, China; wanglan@zju.edu.cn (L.W.); 1194070@zju.edu.cn (Y.Y.); 2 Center for Applied Statistics, School of Statistics, Renmin University of China, Beijing 100872, China; wkh1047925610@ruc.edu.cn (K.W.); wlxu@ruc.edu.cn (W.X.); 3 School of Intelligent Systems Engineering, Sun Yat-sen University, Guangzhou 510006, China; zhongh39@mail2.sysu.edu.cn; 4 Collaborative Innovation Center of Recovery and Reconstruction of Degraded Ecosystem in Wanjiang Basin Co-Founded by Anhui Province and Ministry of Education, School of Ecology and Environment, Anhui Normal University, Wuhu 241002, China; zhaona@ahnu.edu.cn; 5 Department of Infectious Diseases, Zhejiang Provincial Center for Disease Control and Prevention, Hangzhou 310051, China

**Keywords:** respiratory infectious disease incidence, air pollutant concentrations, China, influenza, air quality, Coronavirus disease 2019

## Abstract

The Yangtze River Delta is one of the top five Chinese regions affected by COVID-19, as it is adjacent to Hubei Province, where COVID-19 first emerged. We investigated the impact of COVID-19 non-pharmaceutical interventions (NPIs) on changes in respiratory infectious diseases (RIDs) incidence and air quality in the Yangtze River Delta by constructing two proportional tests and fitting ARIMA and linear regression models. Compared with the pre-COVID-19 period, the average monthly incidence of seven RIDs decreased by 37.80% (*p* < 0.001) and 37.11% (*p* < 0.001) during the COVID-19 period and the post-vaccination period, respectively, in Shanghai, and decreased by 20.39% (*p* < 0.001) and 22.86% (*p* < 0.001), respectively, in Zhejiang. Similarly, compared with the pre-COVID-19 period, the monthly overall concentrations of six air pollutants decreased by 12.7% (*p* = 0.003) and 18.79% (*p* < 0.001) during the COVID-19 period and the post-vaccination period, respectively, in Shanghai, and decreased by 12.85% (*p* = 0.008) and 15.26% (*p* = 0.001), respectively, in Zhejiang. Interestingly, no significant difference in overall incidence of RIDs and concentrations of air quality was shown between the COVID-19 period and the post-vaccination period in either Shanghai or Zhejiang. This study provides additional evidence that the NPIs measures taken to control COVID-19 were effective in improving air quality and reducing the spread of RIDs. However, a direct causal relationship has not been established.

## 1. Introduction

Coronavirus disease 2019 (COVID-19) was first identified in Wuhan, China, in December 2019, and since then the number of COVID-19 cases has rapidly surged worldwide [1]. It was declared a pandemic by the World Health Organization (WHO) on 11 March 2020 [2]. By 5 January 2022, 293,878,679 confirmed cases and 5,454,463 deaths had been reported in more than 225 countries, areas, and territories [3].

In China, the emergence of Severe Acute Respiratory Syndrome Coronavirus-2 (SARS-CoV-2) was first confirmed in Wuhan [4,5]. In January 2020, SARS-CoV-2 spread rapidly to many other cities in Hubei Province and beyond. In response to the threat, the Chinese government implemented numerous strict measures to control SARS-CoV-2 transmission during the initial emergency response to COVID-19 between January and March 2020 [6]. China had controlled its initial COVID-19 epidemic in March 2020, and on 29 April 2020, the country entered the routine control stages of COVID-19 suppression [7]. Although dozens of small COVID-19 outbreaks have occurred in Shijiazhuang in Hebei Province, Beijing, Jilin Province, and the Xinjiang Uygur autonomous region since June 2020 [8,9], these were controlled by continuous containment and suppression [10]. NPIs have included lockdowns, school closures, delayed returns to school, travel restrictions, stay-at-home orders, event bans, quarantines, curfews, and mask mandates. NPIs have also decreased the incidence of common RIDs—including influenza, respiratory syncytial virus, and adenovirus—in New Zealand, the US, Japan, and other countries [11,12,13,14,15]. However, the effect of the measures taken to contain COVID-19 on the incidence of RIDs in China (other than that for COVID-19) remains unclear.

The Yangtze River Delta is one of the top five regions affected by COVID-19 in China, with 1835 and 1322 confirmed cases having been reported in Shanghai Province and Zhejiang Province, respectively, as of 16 March 2021, by local health commissions. The Yangtze River Delta is one of the most densely populated regions on earth and belongs to the more developed areas of China, with over 150 million registered residents. It is characterized by highly sensitive infection disease surveillance, reporting, and detection capabilities. However, a better understanding is required of the effect of the COVID-19 control measures, implemented in 2020, on the incidence of RIDs in Shanghai and Zhejiang.

In 2019, the WHO listed 10 environmental threats to global health; of these, air pollution was considered the greatest threat [16]. Previous studies have demonstrated a positive association between exposure to air pollution and the rate of RIDs [16,17]. For example, recent Chinese studies reported that short-term exposure to PM_2.5_, PM_10_, CO, NO_2_, and O_3_ was significantly associated with confirmed COVID-19 cases [18]. This study aimed to analyze the effect of COVID-19 NPIs measures and COVID-19 vaccinations on the incidence of RIDs and air pollutant concentrations, and assess the association between air pollutant concentrations and the incidence of RIDs from January 2017 to October 2021. We compared the incidence of eight common RIDs (i.e., epidemic parotitis, influenza, measles, pulmonary tuberculosis, rubella, scarlet fever, pertussis, and COVID-19) and six air pollutants (i.e., PM_2.5_, PM_10_, NO_2_, SO_2_, CO, and O_3_) in Shanghai and Zhejiang among three periods: pre-COVID-19, during COVID-19 and post-vaccination. In addition, we attempted to identify potential environmental risks associated with the change in RIDs’ incidence in Shanghai and Zhejiang.

## 2. Materials and Methods

### 2.1. Ethics Statement

This study was conducted according to the principles and guidelines of the Declaration of Helsinki, and was approved by the Research Ethics Committee of the Zhejiang Provincial Center for Disease Control and Prevention (No. 2020-24). All initial information identifying patients was anonymized in this study.

### 2.2. Data Collection (Eight RIDs)

Two data sources—the Shanghai Municipal Health Commission and the Health Commission of Zhejiang Province of China—were used to obtain information on the incidence of the aforementioned eight common RIDs. The data included details—the number of cases, incidence, patient data stratified by onset date (month and year), and area—of probable, clinically diagnosed, and confirmed RID cases. The population data were acquired from the National Bureau of Statistics of the People’s Republic of China, updated at the end of each year.

### 2.3. Data Collection (Air Pollutants)

The average monthly data on the concentrations of the aforementioned six air pollutants in Shanghai and Zhejiang were obtained from an air pollution database in East China, managed by Sun Yat-sen University. The data were obtained from 19 and 56 air quality monitoring stations in Shanghai and Zhejiang, respectively (Supplementary Figure S1).

### 2.4. Case Definitions

The diagnostic criteria of all eight RIDs were issued by the National Health Commission of the People’s Republic of China (Supplementary Table S1 1–7). However, the case definition of seasonal influenza was changed in 2019. A confirmed case of seasonal influenza is defined thus: clinical presentation is that of any of a number of acute febrile respiratory diseases (e.g., fever, cough, coryza, and difficulty breathing) or a history of contact with a confirmed or suspected case of influenza, and a laboratory test positive for the influenza virus, including influenza antigens, PCR, viral isolation, or a fourfold or greater increase in serum antibodies specific for this virus isolated in paired sera.

### 2.5. Statistical Analysis

The monthly and annual incidence of RIDs (per 100,000) were defined as the number of monthly and annual cases of RIDs divided by the population size. To visually demonstrate the impact of COVID-19 on the incidence of RIDs and air pollutant concentrations, the data were categorized according to different periods: 2017–2019 (pre-COVID-19), 2020 (during COVID-19), and January–October 2021 (post-vaccination). Using statistics from two proportional tests with an asymptotic normal distribution, we compared the average yearly incidence of RIDs before and during COVID-19, and we analyzed the changes in the average monthly incidence of RIDs and air pollutant concentrations in the above three stages. We used the seasonal ARIMA model [19] to predict the incidence of RIDs and the concentration of air pollutants during COVID-19 period, and two proportional tests were constructed to check whether the real incidence was the same as the predicted incidence of RIDs in this period. The widely used Pearson correlation coefficient was employed to calculate the association between the incidence of RIDs and air pollutants in Shanghai and Zhejiang. A two-ratio test was performed to determine whether the incidence was equal between the emergency stage and a routine period. A linear regression model was used to identify changes in the incidence data. A *p*-value of less than 0.05 was considered statistically significant. All analyses were conducted using R statistical software. The seasonal ARIMA model was constructed using the function “auto.arima” in the forecast package.

## 3. Results

### 3.1. Descriptive Analysis of the Difference in the Incidence of RIDs between Pre-COVID-19 Period and during COVID-19 Period

**Figure 1:**
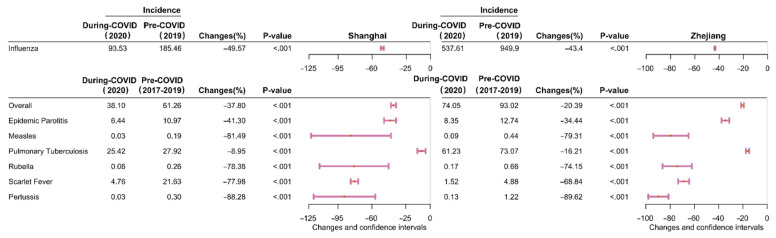
Differences in the average annual incidence of eight respiratory infectious diseases in Shanghai and Zhejiang comparing pre-COVID-19 and COVID-19 periods. Note: The pink lines reflect differences in the 95% confidence interval values. The pink dots indicate the median points. Changes = (x_2_ − x_1_)/x_1_ × 100%, x_1_: average annual incidence of respiratory infectious disease in pre-COVID-19 period, x_2_: average yearly incidence in during COVID-19 period. The *p*-values were computed using two proportional tests.

**Figure 2:**
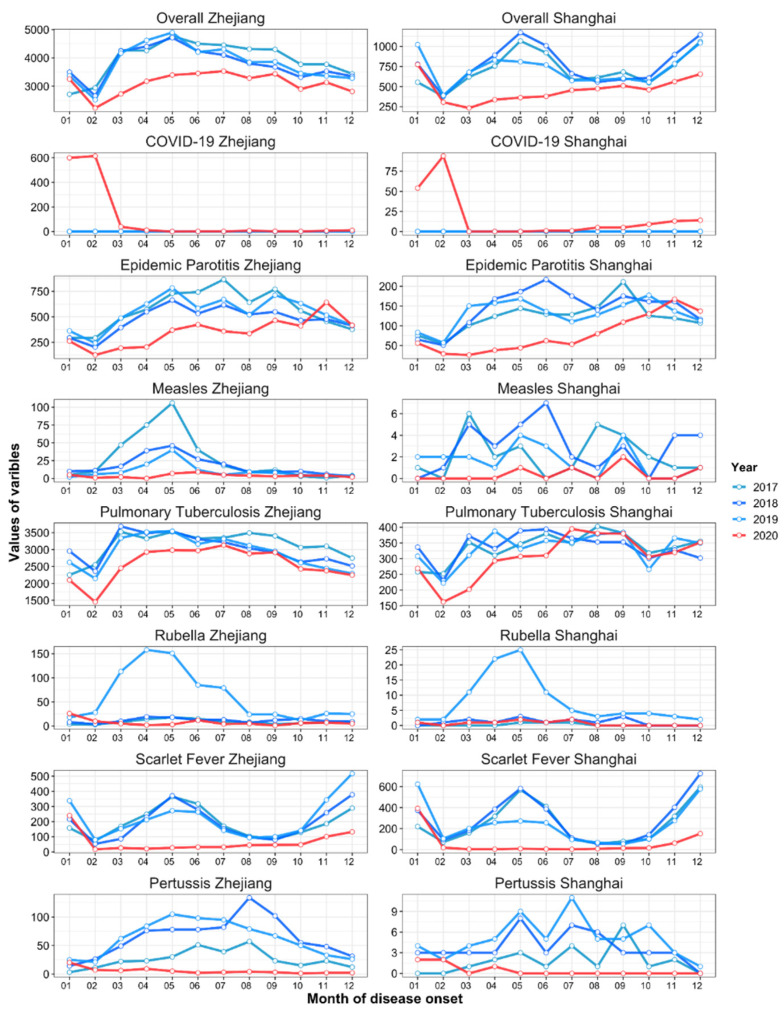
Differences in the monthly cases of seven respiratory infectious diseases in Zhejiang and Shanghai comparing pre-COVID-19 and COVID-19 periods. Note: The horizontal and vertical axes represent the onset months and number of monthly cases, respectively.

**Figure 3:**
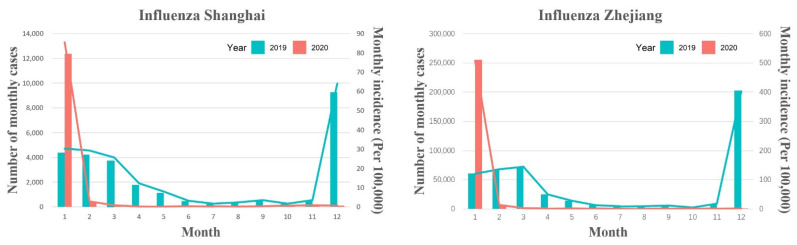
Differences in the monthly cases of influenza in Shanghai and Zhejiang comparing pre-COVID-19 and COVID-19 periods.

Between January 2017 and October 2021, 87,909 and 1,103,808 cases of eight different types of RID were reported in Shanghai and Zhejiang, respectively. The incidence (per 100,000) for seven of the eight RIDs (excepting influenza) decreased by 37.80% (95% confidence interval (CI): 35.15–40.45)—from 61.26 cases in pre-COVID-19 period to 38.10 in during COVID-19 period (*p* < 0.001) in Shanghai (Figure 1 and Figure 2 and Supplementary Table S2), and decreased by 20.39% (95% CI: 19.17–21.61)–from 93.02 cases in pre-COVID-19 period to 74.05 in during COVID-19 period (*p* < 0.001) in Zhejiang (Figure 1 and Figure 2 and Supplementary Table S3). The incidence of influenza decreased by 49.57% (95% CI: 48.10–51.04) and 43.40% (95% CI: 43.05–43.76) in Shanghai and Zhejiang, respectively, between 2019 and 2020 *(p* < 0.001; Figure 3 and Supplementary Tables S2 and S3). Generally, the average annual overall incidence of RIDs during both periods was considerably higher in Zhejiang than Shanghai (Supplementary Table S4).

### 3.2. Descriptive Analysis of the Difference in the Overall Incidence of RIDs during the Emergency and Routine Responses to COVID-19 during the COVID-19 Period

**Figure 4:**
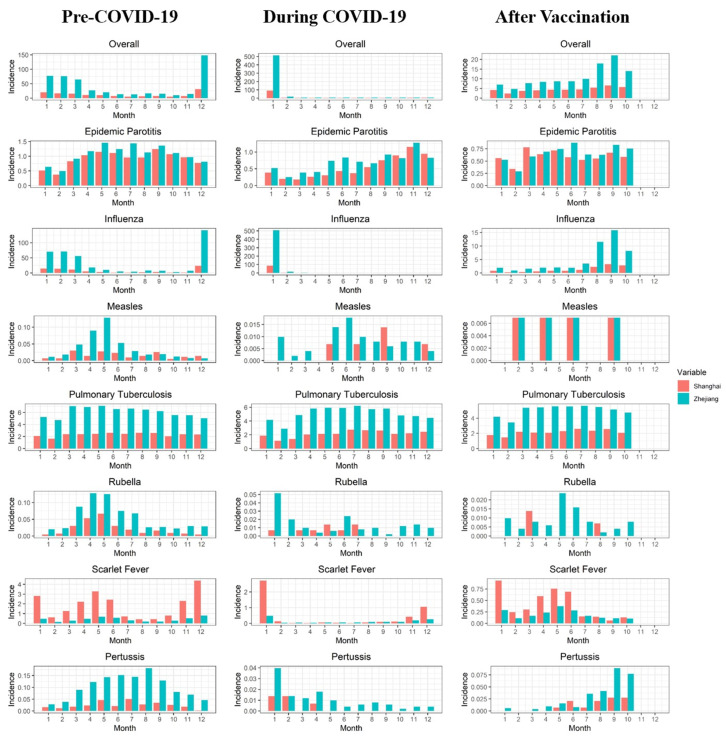
Differences in the monthly average incidence of respiratory infectious diseases in Shanghai and Zhejiang comparing pre-COVID-19, during COVID-19 and post-vaccination periods. Note: The horizontal and vertical axes reflect the onset months and average monthly incidence of RIDs (per 100,000), respectively.

**Figure 5:**
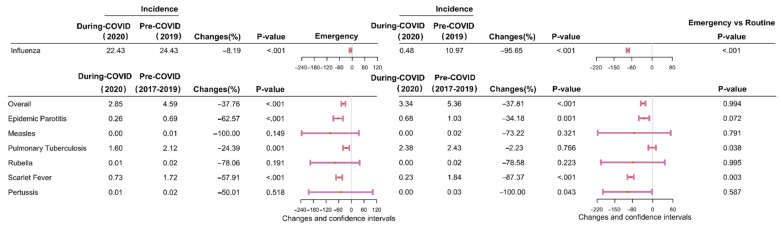
Differences in the average monthly incidence of eight respiratory infectious diseases in the emergency response stage (January to April 2020) and the routine response stage (May to December 2020) in Shanghai. Note: The pink lines reflect differences in 95% confidence interval values. The pink dots indicate the median points. Changes = (x_2_ − x_1_)/x_1_ × 100%, x_1_: average monthly incidence in pre-COVID-19 period, x_2_: average monthly incidence in during COVID-19 period. The *p*-values were computed using two proportional tests. For the emergency or routine response stages, the *p*-values for the emergency versus the routine response stages were computed using two ratio tests.

**Figure 6:**
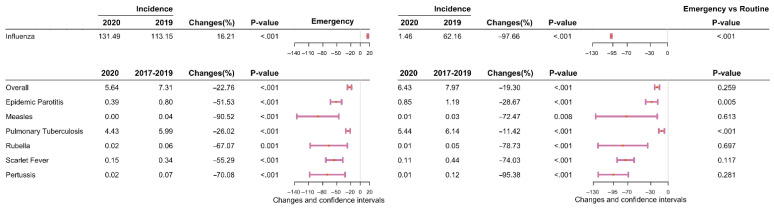
Differences in the average monthly incidence of eight respiratory infectious diseases in the emergency response stage (January to April 2020) and the routine response stage (May to December 2020) in Zhejiang Province. Note: The pink lines reflect differences in 95% confidence interval values. The pink dots indicate the median points. Changes = (x_2_ − x_1_)/x_1_ × 100%, x_1_: average monthly incidence in pre-COVID-19 period, x_2_: average monthly incidence in during COVID-19 period. The *p*-values were computed using two proportional tests. For the emergency or routine response stages, the *p*-values for the emergency versus the routine response stages were computed using two ratio tests.

The monthly overall incidence of RIDs was considerably higher in Zhejiang than in Shanghai for both periods (*p* < 0.050; Figure 4). The COVID-19 period under evaluation was divided into an emergency response stage (January to April 2020) and a routine response stage (May to December 2020). During the emergency response stage, the overall incidence of seven RIDs decreased significantly—by 37.76% in Shanghai (95% CI: 28.07–47.46, *p* < 0.001; Figure 5 and Supplementary Table S5) and by 22.76% in Zhejiang (95% CI: 18.44–27.07, *p* < 0.001; Figure 6 and Supplementary Table S6). During the routine response stage, the incidence of seven of the eight RIDs (excepting influenza) decreased by 37.81% (95% CI: 28.85–46.78) and 19.30% (95% CI: 15.13–23.47) in Shanghai and Zhejiang, respectively (*p* < 0.001; Figure 5 and Figure 6 and Supplementary Tables S5 and S6).

The incidence of influenza decreased significantly in Shanghai in 2020, particularly during the routine stage (95.65% decrease, 95% CI: 90.62–100.68; Supplementary Table S5). However, influenza increased mildly in Zhejiang during the emergency stage (16.21% increase, 95% CI: 15.00–17.41), then decreased by 97.66% during the routine stage (95% CI: 96.54–98.78; Supplementary Table S6).

### 3.3. Descriptive Analysis of the Difference in the Actual and Predicted Overall Incidence of Respiratory Infectious Disease during the COVID-19 Period

**Figure 7:**
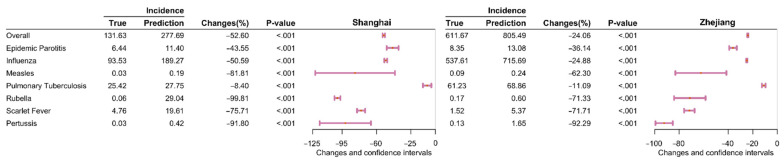
A comparison of the actual and predicted average annual incidence of eight respiratory infectious diseases in Shanghai and Zhejiang Province during the COVID-19 period. Note: The pink lines reflect differences in 95% confidence interval values. The pink dots indicate the median point. The *p*-values were computed using two proportional tests. Changes = (x_2_ − x_1_)/x_1_ × 100%, x_1_: average yearly incidence in 2020 prediction; x_2_: average yearly incidence in 2020.

A seasonal ARIMA model was used to predict the incidence of RIDs and to compare the difference in actual and predicted incidence. In Shanghai, the actual overall incidence of RIDs was 52.60% lower (95% CI: 51.41–53.78, *p* < 0.001) than the predicted incidence (131.63 vs. 277.69 per 100,000; Figure 7 and Supplementary Table S7). Similarly, in Zhejiang, the actual incidence was considerably lower (24.06%) than the predicted incidence (95% CI: 23.66–24.46, 611.67 vs. 805.49 per 100,000, *p* < 0.001; Figure 7 and Supplementary Table S8).

### 3.4. Descriptive Analysis of the Difference in the Average Monthly Overall Incidence of RIDs Comparing Three Periods: Pre-COVID-19, during COVID-19, and Post-Vaccination 

The results of the comparisons of the average monthly incidence comparing pre-COVID-19, during COVID-19 and post-vaccination periods are presented in Supplementary Table S9. In Shanghai, the average monthly overall incidence of RIDs (excepting influenza) decreased significantly during the COVID-19 period (37.80% reduction, *p* < 0.001) and the post-vaccination period (37.11% reduction, *p* < 0.001), compared with the pre-COVID-19 period. In particular, epidemic parotitis (41.30% reduction, *p* < 0.001) and scarlet fever (77.98% reduction, *p* < 0.001) decreased significantly during the COVID-19 period, and they decreased by 34.97% (*p* = 0.002) and 77.78% (*p* < 0.001), respectively, in the post-vaccination period. The overall incidence of RIDs (excepting influenza) showed no significant difference when comparing COVID-19 period post-vaccination periods. The average monthly incidence of influenza during the COVID-19 period and the post-vaccination period decreased by 49.57% (*p* < 0.001) and 91.49% (*p* < 0.001), respectively, compared with 2019, and decreased by 83.12% (*p* < 0.001) in the post-vaccination period compared with the COVID-19 period. In Zhejiang, compared with the pre-COVID-19 period, the average monthly overall incidence of RIDs (excepting influenza) decreased by 20.39% (*p* < 0.001) and 22.86% (*p* < 0.001) during the COVID-19 period and the post-vaccination period, respectively. The average monthly incidence of all seven RIDs (excepting COVID-19) decreased significantly during the COVID-19 period and the post-vaccination period (*p* < 0.05). The overall incidence of RIDs (excepting influenza) showed no significant difference comparing the COVID-19 and post-vaccination periods, but scarlet fever and pertussis increased by 55.42% (*p* = 0.006) and 170.26% (*p* = 0.041), respectively. Similar to Shanghai, the average monthly incidence of influenza in Zhejiang during the COVID-19 period and the post-vaccination period decreased by 43.40% (*p* < 0.001) and 93.77% (*p* < 0.001), respectively, compared with 2019, and decreased by 89.00% (*p* < 0.001) in the post-vaccination period compared with the COVID-19 period.

### 3.5. Descriptive Analysis of the Difference in Air Pollutant Concentrations Comparing Three Periods: Pre-COVID-19, during COVID-19 and Post-Vaccination

**Figure 8:**
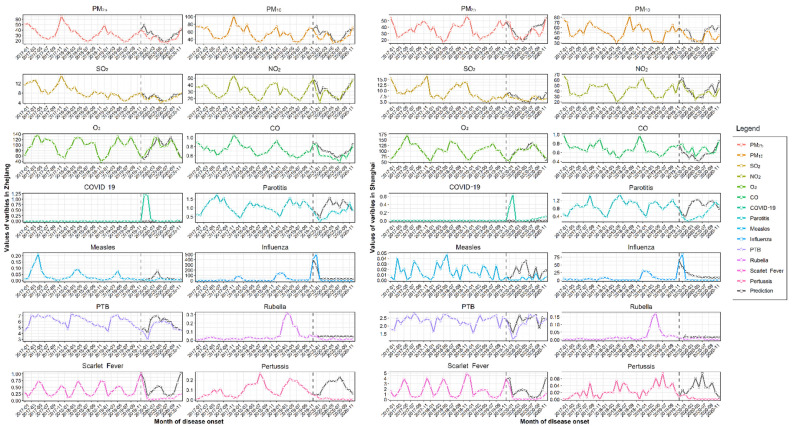
Actual monthly concentrations of six air pollutants and eight respiratory infectious diseases in Zhejiang and Shanghai from 2017–2020 and hypothetical values for 2020 in the absence of COVID-19. Note: The horizontal axis depicts the months. The concentration of pollutants—SO_2_ (μg/m^3^), NO_2_ (μg/m^3^), CO (mg/m^3^), O_3_ (μg/m^3^), PM_2.5_ (μg/m^3^), and PM_10_ (μg/m^3^)—are plotted along the six vertical axes of the first six graphs, and incidence (per 100,000) is plotted along the vertical axes of the last eight graphs. The black lines represent the assumed values of the diseases and pollutants, and the colored lines represent the real values.

The monthly concentrations of the six air pollutants (i.e., PM_2.5_, PM_10_, NO_2_, SO_2_, CO, and O_3_) decreased by 12.66% (95% CI: 4.82–20.49, *p* = 0.003) and 12.85% (95% CI: 3.80–21.89, *p* = 0.008) in Shanghai and Zhejiang, respectively, during the COVID-19 period compared with the pre-COVID-19 period (Supplementary Table S10). The most rapid decrease was in SO_2_ concentrations, which decreased by 32.40% in Shanghai (95% CI: 20.62–44.18) and by 33.36% in Zhejiang (95% CI: 21.20–45.52) (*p* < 0.001; Supplementary Table S10). The next largest decrease was observed in PM_2.5_ concentrations, which fell 26.30% in Zhejiang (95% CI: 8.97–43.62, *p* = 0.004), followed by PM_10_ concentrations in Shanghai (22.39% decrease, 95% CI: 10.23–34.54, *p* < 0.001), PM_10_ concentrations in Zhejiang (19.82% decrease, 95% CI: 4.21–35.43, *p* = 0.015), and PM_2.5_ concentrations in Shanghai (12.80% decrease, 95% CI: −32.84–7.24, *p* ≥ 0.050; Figure 8 and Supplementary Table S10). By comparing the monthly concentrations of air pollutants in the post-vaccination period with those in the pre-COVID-19 period, we found that the six air pollutants decreased significantly in both Shanghai and Zhejiang—by 18.54% (95% CI: 9.83–27.26, *p* < 0.001) and 15.26% (95% CI: 6.85–23.68, *p* = 0.001), respectively. In Shanghai, SO_2_ decreased the most (40.76%, 95% CI: 28.06–53.46, *p* < 0.001), followed by PM_2.5_ (29.90%, 95% CI: 11.55–48.25, *p* = 0.003). In Zhejiang, PM_2.5_ decreased the most (34,41%, 95% CI: 16.17–52.65, *p* < 0.001), followed by SO_2_ (32.45%, 95% CI: 20.17–44.73, *p* < 0.001). No significant difference was found with regard to air pollutant concentrations when comparing the COVID-19 period and the post-vaccination period in either Shanghai of Zhejiang.

### 3.6. Descriptive Analysis of the Relationship between the Incidence of RIDs and Air Pollutant Concentrations

**Figure 9:**
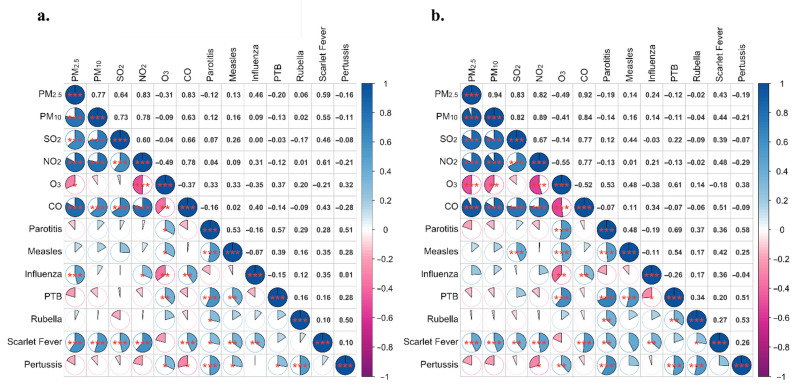
Pearson’s correlation coefficients for air pollution concentrations and the incidence of seven respiratory infectious diseases in Shanghai and Zhejiang between 2017 and 2021. Note: (**a**): the data from Shanghai, (**b**): the data from Zhejiang. PM_2.5_: atmospheric particulate matter (PM) with a diameter of less than 2.5 μm, PM_10_: PM with a diameter of less than 10 μm, SO_2_: sulfur dioxide, NO_2_: nitrogen dioxide, O_3_: ozone, CO: carbon monoxide. * *p*-value < 0.05, ** *p*-value < 0.01, *** *p*-value < 0.001. PTB: Pulmonary tuberculosis.

Based on the incidence of RIDs and pollutant concentrations in Shanghai and Zhejiang from 2017 to October 2021, we found that the overall incidence of seven RIDs in both Shanghai and Zhejiang was related to O_3_ (*p* < 0.05); it also correlated with NO_2_ and PM_2.5_ in Shanghai (*p* < 0.05) and CO in Zhejiang (*p* < 0.05). A moderately positive correlation was identified between the incidence of influenza and monthly PM_2.5_ concentrations (*r* = 0.46), CO concentrations (*r* = 0.40), and NO_2_ concentrations (*r* = 0.31) in Shanghai, as well as CO concentrations (*r* = 0.34) in Zhejiang, when evaluated using Pearson correlation (*p* < 0.050; Figure 9). The negative association between O_3_ and influenza incidence was found both in Shanghai (*r* = −0.35) and Zhejiang (*r* = −0.38; Figure 9).

**Figure 10:**
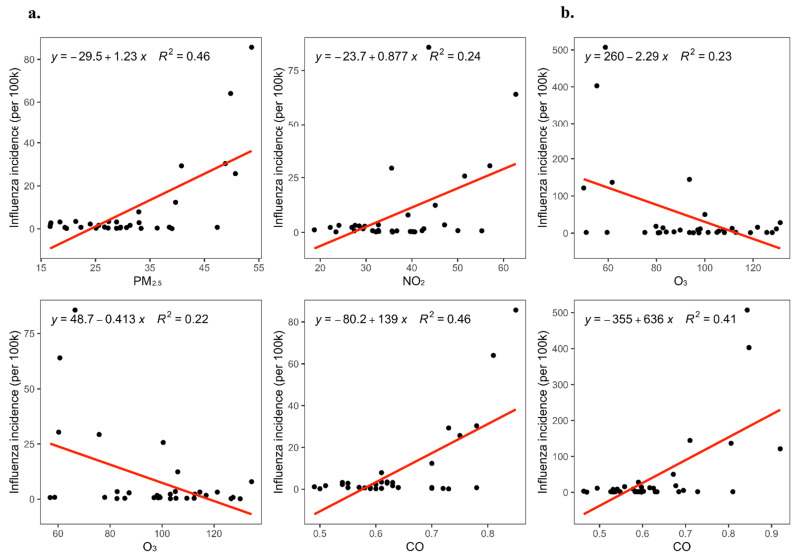
Linear regression of air pollution concentrations on influenza incidence in Shanghai and Zhejiang. Note: (**a**): The effect of PM_2.5_, NO_2_, O_3_, and CO concentrations on influenza incidence in Shanghai using linear regression. (**b**): The effect of O_3_ and CO concentrations on influenza incidence in Zhejiang using linear regression.

We used linear regression models to determine the impact of air pollutant concentrations on the incidence of influenza. The results showed that CO, NO_2_, and PM_2.5_ concentrations were positively associated with influenza incidence in Shanghai. However, O_3_ concentrations were found to negatively correlate with influenza incidence (Figure 10a). In Zhejiang, CO concentrations were positively correlated with influenza incidence. Conversely, O_3_ concentrations were negatively correlated with influenza incidence (Figure 10b).

## 4. Discussion

Using representative data from the Yangtze River Delta, the current study identified a marked decline in the annual overall incidence of eight RIDs during the COVID-19 period and the post-vaccination period compared with the pre-COVID-19 period. The overall incidence of RIDs during the COVID-19 period was lower than its predicted incidence in both regions. A similar decrease was demonstrated in the concentrations of six air pollutants during the COVID-19 period and the post-vaccination period; of these, PM_2.5_, O_3_, and CO were significantly associated with a decrease in influenza incidence.

Because Shanghai and Zhejiang are geographically proximal to Hubei Province, a large proportion of the population returned from Hubei Province before Wuhan went into lockdown on 23 January 2020. Both areas were the first to be impacted by COVID-19 [5]. Zhejiang and Shanghai reported the first imported cases of COVID-19 (from Hubei Province), on 20 and 23 January, respectively [20]. Thereafter, COVID-19 spread rapidly over the next two months, to 11 cities in Zhejiang Province and to 16 Shanghai districts. In response to this novel threat, on 23 January the governor initiated a top-level emergency response to COVID-19 for implementation in Zhejiang and Shanghai. The application of strict precautionary measures, such as lockdowns, home stays, school closures, and the suspension of large-scale events, disrupted the transmission of COVID-19, which simultaneously resulted in a decrease in the incidence of other RIDs, including influenza. In this regard, the findings of the current study support those of studies conducted outside China [12,13,14,21]. Several explanations have been posited for the decrease in the incidence of RIDs. First, these diseases have a similar mode of transmission to COVID-19 (i.e., via respiratory transmission or contact) [12,13]; therefore, it may be assumed that interventions to prevent COVID-19 have reduced the spread of RIDs. Second, difficulty accessing hospital services during COVID-19—owing to strict quarantine measures, together with fears of contracting COVID-19 at a hospital—have inhibited opportunities for disease transmission [11]. Last, people’s hygiene habits have improved considerably, as it is mandatory to wear masks, regularly wash hands, and implement proper ventilation. Generally, it is feasible that the lockdown, in conjunction with health-seeking behavioral changes and improved personal hygiene, has reduced the risk of the transmission of RID pathogens [10,11,12,13,14,21].

In the current study, the rate of influenza slightly increased during the emergency response stage (during COVID-19) in Zhejiang, which may be due to the public’s increased awareness of the similarities between RIDs and COVID-19 symptoms, together with enhanced surveillance, testing, and strategies to diagnose influenza-like illnesses in early 2020. However, the incidence of influenza remained significantly low during the routine response stage (during COVID-19) in both regions. In particular, the average monthly incidence of influenza decreased significantly in the post-vaccination period, compared with pre-COVID-19 and COVID-19 periods. This finding could be attributed to the efficacy of COVID-19 prevention strategies, and the vaccine may also have had an impact on reducing the incidence of influenza.

COVID-19 prompted a period of nationwide public lockdown, which provided an invaluable opportunity for an evaluation of the correlation between air pollutant concentrations and RIDs. In the current study, the monthly concentrations of six air pollutants were found to have decreased significantly during the COVID-19 period and the post-vaccination period, compared with the previous three years, and the levels were much lower than those stipulated in the Chinese guidelines [16]. In the COVID-19 period and the post-vaccination period, this reduction can be attributed to the reduction of primary air pollutant emissions. For example, almost all medium and small industries (excepting power plants and large-scale enterprises) were closed [22]. In addition, officially, the Yangtze River Delta cities entered full lockdown between January 23 and 25 and remained locked down until the end of April. These policies led to reduced human activity, which improved air quality as a byproduct. Similar findings were reported elsewhere in this regard [22]. There was no significant difference in air pollutant concentrations during the COVID-19 period and the post-vaccination period, which may be mainly attributed to three reasons. Firstly, the Shanghai and Zhejiang government pursued a strong elimination strategy, no large-scale outbreaks of the epidemic were found in Shanghai and Zhejiang, and the small-scale outbreaks of the epidemic were quickly controlled. Secondly, there was no large-scale suspension of production during the COVID-19 period and the post-vaccination period, due to the rapid elimination strategy and less interventions in production. Thirdly, all non-pharmaceutical interventions, including control gathering, travel limitation, and increased social distance, etc. are still being further strengthened in the routine stages (after 30 April 2020).

We were motivated to determine if low air pollutant concentrations were associated with the decrease in the incidence of RIDs. As with previous findings, it was established that the decrease in people’s short-term exposure to air pollutants inhibited the spread of RIDs in the Yangtze River Delta. Specifically, PM_2.5_, CO, and NO_2_ concentrations in Shanghai and CO concentrations in Zhejiang significantly affected the incidence of influenza.

The reasons for this are unclear but may involve the differences between primary emissions, population density, and energy and industrial strategies. First, a recent epidemiological study concluded that decreased short-term exposure to particulate matter was associated with a decline in the use of healthcare services for acute lower respiratory infections [23]. Second, exposure to urban airborne particulate matter has been demonstrated to alter the macrophage-mediated inflammatory response to respiratory viral infection [18,23]. Third, exposure to PM_10_ and PM_2.5_ could significantly increase RNA virus infections, such as H1N1 and H5N1, in A459 human lung epithelial cells by increasing viral replications [16,24]. Fourth, air pollutants affect the lower respiratory tract’s protease–antiprotease balance and microflora, which are associated with respiratory infections [18].

To the best of our knowledge, this is the first study to have evaluated differences in the incidence of RIDs and air pollutant concentrations in the Yangtze River Delta in the pre-COVID-19 period, during COVID-19, and the post-vaccination period. However, the study has several limitations. First, the evaluation was limited to air pollutants; it is possible that other meteorological factors might have influenced the transmission and pathogenesis of respiratory infections. Second, this study might feature reporting biases because of increased healthcare-seeking behavior and decreased laboratory capacity. Third, this is an ecological study, and we identify the decline of RIDs and the concentrations of six air pollutants. However, current evidence cannot prove a causal relationship between air pollution and RIDs.

## 5. Conclusions

The current study indicates that the incidence of eight RIDs and the concentration of six air pollutants in Shanghai and Zhejiang decreased significantly during the COVID-19, and the post-vaccination period compared with the pre-COVID-19 period. The most rapid decrease was observed in influenza. However, direct causality is not established between the changes in air pollutant concentrations and the incidence of overall respiratory diseases. In contrast, the influenza reduction was positively correlated with NO_2_, CO, and PM_2.5_ concentrations in Shanghai but associated with CO concentrations in Zhejiang. No significant difference in overall incidence of RIDs and the concentrations of air quality was shown when comparing the COVID-19 period and the post-vaccination period in either Shanghai or Zhejiang. This study provides additional evidence that the measures taken to combat COVID-19 were effective in greatly improving air quality and decreased RIDs. In future studies, the exact mechanisms of these effects should be explored further using a large-sample design; more stringent regional joint control in the Yangtze River Delta should be advanced to improve air quality and infectious disease control.

## Data Availability

Publicly available datasets were analyzed in this study. The data can be found here: monthly reported cases of notifiable infectious diseases in Shanghai and Zhejiang: Shanghai municipal health commission (https://wsjkw.sh.gov.cn/yqxx/index.html), health commission of Zhejiang Province (https://wsjkw.zj.gov.cn/col/col1229123469/index.html); daily air pollutant concentration data in Shanghai and Zhejiang: real-time monitoring of air quality in cities across China (http://106.37.208.233:20035/).

## Supplementary Materials

The following supporting information can be downloaded at: www.mdpi.com/article/10.3390/ijerph19031286/s1, Figure S1: The geographical location of air quality monitoring stations from 2017–2020 in Shanghai and Zhejiang Province, China; Table S1-1: The diagnostic criteria for pulmonary tuberculosis in China; Table S1-2: The diagnostic criteria for influenza in China; Table S1-3: The diagnostic criteria for measles in China; Table S1-4: The diagnostic criteria for mumps in China; Table S1-5: The diagnostic criteria for rubella in China; Table S1-6: The diagnostic criteria for pertussis in China; Table S1-7: The diagnostic criteria for scarlet fever in China; Table S2: Changes in the average yearly incidence rates of 8 respiratory infectious diseases in Shanghai, China; Table S3: Changes in the average yearly incidence rates of 8 respiratory infectious diseases in Zhejiang Province, China; Table S4: Comparison of the average yearly incidence rates of 8 respiratory infectious diseases between Shanghai and Zhejiang, China; Table S5: Changes in the average monthly incidence rates of 8 respiratory infectious diseases in the emergency response stage (January to April 2020) and the routine response stage (May to December 2020) in Shanghai, China; Table S6: Changes in the average monthly incidence rates of 8 respiratory infectious diseases in the emergency response stage (January to April 2020) and the routine response stage (May to December 2020) compared with the previous three years in Zhejiang Province, China; Table S7: Comparison of the actual and predicted average incidence rates of 8 respiratory infectious diseases in Shanghai in 2020; Table S8: Comparison of the true and predicted average incidence rates of 8 respiratory infectious diseases in Zhejiang Province in 2020; Table S9: Comparisons of average monthly incidence of respiratory infectious diseases among 2021, 2020, and 2017–2019; Table S10: Changes in average monthly concentration of 6 air pollutants among 2021, 2020, and 2017–2019 in Shanghai and Zhejiang Province, China.
