# Supplementary material for: The Effect of Coronavirus 2019 Disease Control Measures on the Incidence of Respiratory Infectious Disease and Air Pollutant Concentrations in the Yangtze River Delta Region, China
Source: Int J Environ Res Public Health. 2022 Jan 24;19(3):1286. doi: 10.3390/ijerph19031286 (PMC8835036; doi:10.3390/ijerph19031286)
Supplement: Supplementary file 1 [file ijerph-19-01286-s001.zip › ijerph-1485699-supplementary.pdf]

Supplementary Files

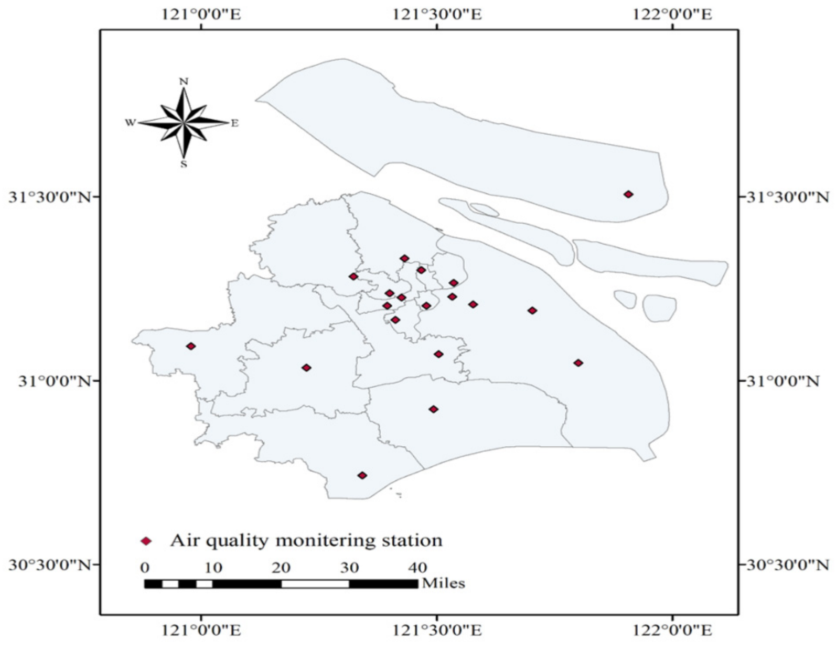

1a

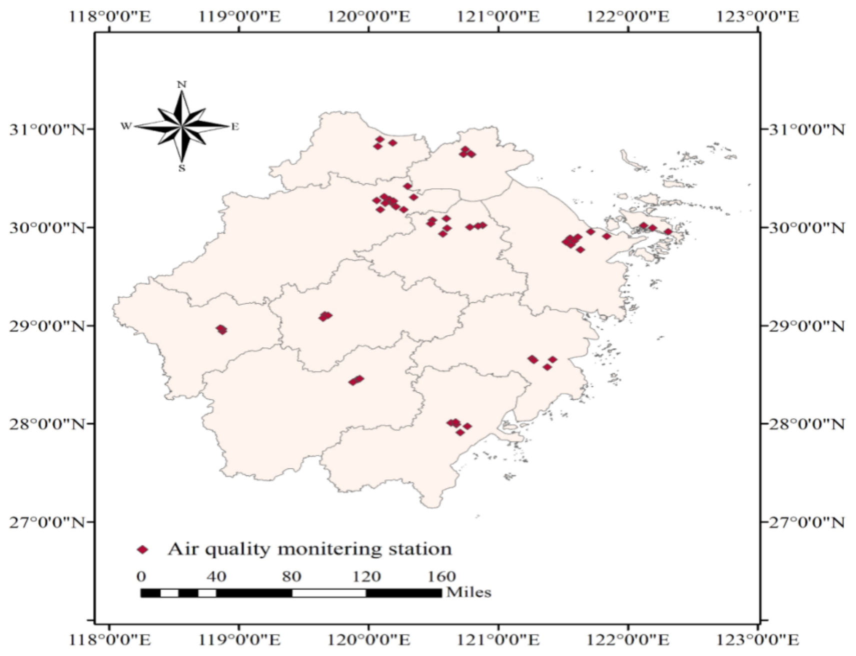

1b

Supplementary Figure S1. The geographical location of air quality monitoring stations from 2017–2020 in Shanghai and Zhejiang Province, China

Notes: 1a: Air quality monitoring stations in Shanghai (n = 19); 1b: Air quality monitoring stations in Zhejiang Province (n = 56)

Supplementary Table S1-1: The diagnostic criteria for pulmonary tuberculosis in China

Standard Name	Diagnostic Criteria of Pulmonary Tuberculosis
**1 Scope**	This standard is applicable to the diagnosis of tuberculosis in all kinds of medical and health institutions and their medical personnel at all levels in the country.
**2 Terms and definitions**	2.1 Pulmonary tuberculosis<br>2.2 Mycobacterium tuberculosis
**3 Diagnostic basis**	
**3.1 Epidemiologic Linkage**	The patient has an epidemiologic history of contact with acute active pulmonary tuberculosis
**3.2 Clinical manifestations**	3.2.1 Symptoms: Cough, sputum or blood or haemoptysis in sputum lasting longer than two weeks
	3.2.2 Physical examination: A physical examination is done to assess the patient's general health. It cannot be used to confirm or rule out TB. However, certain findings are suggestive of TB; for example, blood in the sputum, significant weight loss and drenching wet rales, wheezing sound, superficial lymphadenopathy, and night sweats may be due to TB.
**3.3 Chest Imaging Examination**	3.3.1 Primary pulmonary tuberculosis: Primary pulmonary lesions and intrathoracic lymphadenopathy, or simple intrathoracic lymphadenopathy;<br>3.3.2 Haematogenous disseminated pulmonary tuberculosis: Miliary shadow with uniform size and density in both lungs;<br>3.3.3 Secondary tuberculosis: The chest imaging manifestations of secondary pulmonary tuberculosis are diverse. In light cases, patches, nodules and striations are the main manifestations, or tuberculoma or solitary voids; in heavy cases, lobar infiltration, caseous pneumonia, multiple void formation and bronchial dissemination are the main manifestations; in repeated delays of progression, pulmonary lesions may occur, the volume of damaged lung tissue

	is reduced, multiple thick-walled fibrous holes, secondary bronchiectasis, or multiple calcification are the main manifestations. Pulmonary hilum and mediastinum structure show traction displacement, thoracic collapse, pleural thickening and adhesion; other lung tissues show compensatory emphysema and new and old bronchial disseminated lesions;<br>3.3.4 Tuberculosis of trachea and bronchi: The main manifestations of tracheobronchial tuberculosis are irregular thickening of the trachea or bronchial wall, stenosis or obstruction of the lumen, secondary atelectasis or consolidation, bronchiectasis, and other bronchial disseminated lesions in the distal lung tissue of the stenosed bronchus;<br>3.3.5 Tuberculous Pleurisy: Tuberculous pleurisy is divided into dry pleurisy and exudative pleurisy. Dry pleurisy is an early inflammatory response of the pleura, usually without obvious imaging manifestations; exudative pleurisy is mainly manifested as pleural effusion; and pleural effusion can be manifested as a small or medium amount of free effusion, or a limited effusion in any part of the pleura. Those patients with slow absorption are often accompanied by pleural thickening and adhesion, and can also evolve into pleural tuberculoma and empyema.
**3.4 Laboratory Tests**	3.4.1 Bacteriological test: (a) Positive for *Mycobacterium tuberculosis* by smear microscopy; (b) Mycobacterium was positive in culture and identified as *Mycobacterium tuberculosis* complex.<br>3.4.2 Molecular Biology Examination: Positive nucleic acid for *Mycobacterium tuberculosis*<br>3.4.3 Pathological examination of tuberculosis: Histopathological changes typical of tuberculosis<br>3.4.4 Immunological examination<br>3.4.4.1 Moderate or strong positive tuberculin skin test;<br>3.4.4.2 Positive IFN-gamma release test;<br>3.4.4.3 Positive *Mycobacterium tuberculosis* antibody test.<br>3.4.5 Bronchoscopy: Bronchoscopy can directly reveal the pathological changes in the trachea and bronchus, as well as allow collection of aspirate secretions, brush samples and biopsies.
**4. Diagnostic principles**	The diagnosis of pulmonary tuberculosis is based on etiology (including bacteriology and molecular biology), combined with epidemiological history, clinical manifestations, chest imaging, related auxiliary examinations and differential diagnosis; together, these provide a comprehensive diagnosis. Pathogenic and pathological results are

	used as the basis for confirmation. In the diagnosis of pulmonary tuberculosis in children, besides a sputum etiology examination, young patients should also undergo a gastric etiology examination.
**5. Diagnosis and Classification**	
**5.1 Suspected cases**	Those who meet one of the following criteria:<br>(a) having any of the 3.3 items;<br>(b) Children under 5 years old with 3.2 and 3.1, 3.4.4.1 and 3.4.4.2.
**5.2 Clinically diagnosed cases**	Other pulmonary diseases are excluded by differential diagnosis, and one of the following criteria is met:<br>(a) with any of 3.3 and 3.2;<br>(b) with any of 3.3 and 3.4.4.1;<br>(c) with any of 3.3 and 3.4.4.2;<br>(d) with any of 3.3 and 3.4.4.3;<br>(e) with any of the 3.3 items and with extrapulmonary pathological examination confirming tuberculosis lesions;<br>(f) Tracheal and bronchial tuberculosis are diagnosed with 3.3.4 and 3.5.
**5.3 Confirmed cases**	5.3.1 Diagnosis of sputum smear positive tuberculosis<br>Those who meet one of the following criteria:<br>(a) 2 sputum smears with acid-fast bacilli conform to 3.4.1.a;<br>(b) The acid-fast bacillus test of one sputum specimen conforms to 3.4.1.a, with any of the 3.3 items concomitantly;<br>(c) The acid-fast bacilli test of one sputum specimen is 3.4.1.a, and the culture of *Mycobacterium* in one sputum specimen is 3.4.1.b.<br>5.3.2 Diagnosis of *Mycobacterium* tuberculosis<br>In accordance with any of 3.3, at least 2 sputum smears are negative, and mycobacterium culture agrees with 3.4.1.b.<br>5.3.3 Molecular Biology Examination Positive Diagnosis of Tuberculosis<br>Compliance with either 3.3 or 3.4.2.<br>5.3.4 Diagnosis of Pulmonary Tuberculosis Positive by Histopathological Examination of Lung

	Compliance with 3.4.3.<br>5.3.5 Diagnosis of Tracheobronchial Tuberculosis<br>Those who meet one of the following criteria:<br>(a) Those with 3.5 and 3.4.3 coincidence in pathological examination of trachea and bronchus;<br>(b) Those with 3.5 and 3.4.1.a or 3.4.1.b or 3.4.2 of tracheal and bronchial secretions.<br>5.3.6 Diagnosis of tuberculous pleurisy<br>Those who meet one of the following criteria:<br>(a) Those with 3.3 pleural effusion or pleural pathology accorded with 3.4.3;<br>(b) Those with 3.3 and 3.4.1.a or 3.4.1.b or 3.4.2 of pleural effusion etiology.
**Chinese Standard Number**	WS 288-2017
**Issued by**	National Health Commission of the People’s Republic of China
**Date issued**	November 9, 2017
**Official Source**	http://www.nhc.gov.cn/ewebeditor/uploadfile/2017/11/20171128164254246.pdf

Supplementary Table S1-2: The diagnostic criteria for influenza in China

Standard Name	Diagnostic Criteria of Influenza
**1 Scope**	This standard is applicable to all levels of medical and health institutions and personnel in the country for the diagnosis and reporting of influenza.
**2 Terms and definitions**	Type of influenza virus; Subtype of influenza A virus; Influenza-like illness
**3 Diagnostic basis**	
**3.1 Epidemiologic Linkage**	In the local epidemic season (i.e. winter and spring in northern China and winter, spring and summer in southern China), a large number of upper respiratory tract infections occur in a unit or area, or the number of patients with upper respiratory tract infections in hospital outpatient and emergency departments increases significantly.
**3.2 Clinical manifestations**	3.2.1 The common manifestations are acute fever (axillary body temperature (> 38 C), chills, headache, dizziness, soreness, fatigue and other toxic symptoms, as well as respiratory symptoms, such as pharyngalgia and dry cough, but the catarrhal symptoms are often not obvious.<br>3.2.2 A few cases can have anorexia accompanied by gastrointestinal symptoms, such as abdominal pain, abdominal distention, vomiting and diarrhoea.<br>3.2.3 A few cases can also be complicated with sinusitis, otitis media, laryngitis, bronchitis, pneumonia, and even respiratory and circulatory failure and death.<br>3.2.4 Children under two years of age, or those with chronic underlying diseases, may have lower respiratory sounds and wet beeps or wheezing sounds in both lungs, but no signs of lung consolidation.<br>3.2.5 Thoracic X-ray examination of severe patients can show unilateral or bilateral pulmonary parenchymal lesions, and a few lesions may be accompanied by pleural effusion.<br>3.2.6 The total number of white blood cells in peripheral haemogram is neither high nor low, and the lymphocytes increase relatively. The total numbers of white blood cells and lymphocyte decrease in severe patients.

**3.3 Laboratory Tests**	The procedures for collecting, transporting and treating influenza specimens can be found in Appendix G.<br>3.3.1 Influenza viruses are isolated and identified from patients' respiratory tract specimens (see Appendix A).<br>3.3.2 The titre of serum anti-influenza virus antibody is 4 or more times higher in convalescent patients than in acute patients (see Appendix B and C).<br>3.3.3 Influenza virus specific nucleic acid (see Appendix D) or specific antigen (see Appendix E) is detected in the patients' respiratory tract specimens.<br>3.3.4 After the virus proliferates in sensitive cells for one generation, the specific nucleic acid of influenza virus gives a positive test (see Appendix D for details) or a specific antigen is detected (see Appendix E).
**4. Diagnostic principles**	If only clinical manifestations in a non-epidemic season are considered, distinguishing influenza virus from other pathogens is difficult, especially for diseases caused by other respiratory pathogens. The diagnosis of influenza cases often requires laboratory diagnosis. However, in the flu season, when a large number of patients with upper respiratory tract infections or outpatient and emergency upper respiratory tract infections in a local unit or local area increase significantly, the patients with corresponding clinical manifestations can be considered clinically diagnostic cases of influenza. See Appendix F for the etiology, epidemiology and clinical manifestations of influenza.
**5. Diagnosis and Classification**	
**5.1 Clinically diagnosed cases**	Those with any of the clinical manifestations in 3.1 and 3.2.
**5.2 Confirmed cases**	5.2.1 Influenza-like cases with any of 3.3.<br>5.2.2 Clinically diagnosed cases with any of 3.3.
**Chinese Standard Number**	WS285-2008

**Issued by**	National Health Commission of the People's Republic of China
**Date issued**	February 28, 2008
**Official Source**	

Supplementary Table S1-3: The diagnostic criteria for measles in China

Standard Name	Diagnostic Criteria of Measles
**1 Scope**	This standard is applicable to the diagnosis of measles by medical and health institutions at all levels and their medical personnel
**2 Terms and definitions**	CPE ： cytopathic effect<br><br>DEPC： diethyl pyrocarbonate<br><br>ELISA ： enzyme-linked immunosorbent assay<br><br>IgG： immunoglobulin G<br><br>IgM： immunoglobulin M<br><br>RF： rheumatoid factor<br><br>RNA： ribonucleic acid<br><br>RT-PCR： reverse transcription-polymerase chain reaction<br><br>RPM： revolutions per minute<br><br>OD： optical density value

	VTM： virus transportation medium
**3 Diagnostic basis**	
**3.1 Epidemiologic Linkage**	3.1.1 At 7-21 days before the onset of measles, a patient with measles had contact history.<br>3.1.2 The patient had a history of residence or travel in measles-endemic areas 7 to 21 days before the onset of measles.
**3.2 Clinical manifestations**	3.2.1 Fever, body temperature generally (> 38 C).<br>3.2.2 Red macular papules appear on the 3rd to 4th day of the course of the disease, and the skin between the rashes is normal. The order of eruption usually starts from the back of the ear and the face and extends from top to bottom to the whole body; it can involve the mucosa. The eruption lasts for 3 to 5 days.<br>3.2.3 Upper respiratory catarrhal symptoms, such as cough, runny nose and sneezing, as well as photophobia, tearing and conjunctivitis.<br>3.2.4 Measles mucosal plaques (Koplik plaques) are found on the oral and buccal mucosa at the early stage of onset (usually on the 2nd to 3rd day of the course).
**3.3 Laboratory Tests**	3.3.1 No live attenuated measles vaccine containing measles components were inoculated within 8 to 56 days before blood collection, but measles IgM is positive in blood samples within 28 days after measles emergence<br>3.3.2 Measles virus nucleic acid is positive in throat swabs or urine samples or is isolated from measles virus<br>3.3.3 The titre of measles IgG antibody increases more than 4 times in convalescent blood samples than in acute stage, or the antibody test is negative in the acute stage and positive in the convalescent stage.
**4. Diagnostic principles**	Diagnosis is made according to epidemiological history, clinical manifestations and laboratory results.
**5. Diagnosis and Classification**	
**5.1 Suspected cases**	The patient has 3.2.1, 3.2.2 and 3.2.3.

**5.2 Clinically diagnosed cases**	A suspected case meets any of the following criteria:<br>(a) Has 3.1.1 and/or 3.1.2, and has not been clearly diagnosed as other diseases;<br>(b) 3.2.4;<br>(c) No specimens were collected for laboratory testing, but no other diseases were definitely diagnosed.
**5.3 Confirmed cases**	The suspected cases had any one of 3.3.1, 3.3.2 and 3.3.3.
**Chinese Standard Number**	WS 296-2017
**Issued by**	National Health Commission of the People's Republic of China
**Date issued**	July 24, 2017
**Official Source**	http://www.nhc.gov.cn/ewebeditor/uploadfile/2017/07/20170727145913239.pdf

Supplementary Table S1-4: The diagnostic criteria for mumps in China

Standard Name	Diagnostic Criteria of Mumps
**1 Scope**	This standard is applicable to the diagnosis and reporting of mumps in all kinds of medical and health institutions and their staff at all levels in China.
**2 Diagnostic basis**	
**2.1 Epidemiologic Linkage**	The patient has a history of contact with patients with mumps or an epidemic of mumps has occurred in the area from 14 to 28 days before the onset of the disease.
**2.2 Clinical manifestations**	2.2.1 Fever, headache, fatigue, loss of appetite, etc.<br>2.2. Swelling and pain of the unilateral or bilateral parotid glands and/or other salivary glands are aggravated when opening the mouth, chewing or eating acidic foods.<br>2.2.3 Headache, vomiting, meningeal irritation or changes of consciousness occur in patients with meningoencephalitis.<br>2.2.4 When accompanied by orchitis, swelling and pain occurs in the testis or epididymis.<br>2.2.5 Patients with pancreatitis show vomiting, upper and middle abdominal pain and tenderness.
**2.3 Laboratory Tests**	2.3.1 Leukocyte counts and routine urine tests are normal; leucocyte counts can be increased in patients with orchitis.<br>2.3.2 Most (90%) patients have increased serum and urinary amylase in the early stage of onset. In patients with meningoencephalitis without parotid gland enlargement, amylase can also be elevated in the blood and urine. The increase in serum lipase is helpful in the diagnosis of pancreatitis.<br>2.3.3 About half of the patients may have cerebrospinal fluid changes in viral meningoencephalitis.<br>2. 3. 4 The mumps virus specific IgM antibody is detected in the serum of patients who had not been vaccinated with live attenuated vaccine in the past month.<br>2.3.5 The titre ratio of mumps virus IgG antibody in convalescent and acute sera (interval 2 to 4 weeks) is 4 or more

	times higher (including positive antibody conversion).<br>2.3.6 Mumps virus is isolated from saliva, urine, cerebrospinal fluid and other body fluids (Appendix A.2 Mumps virus can be isolated by any method).
**3. Diagnostic principles**	The diagnosis of parotid gland enlargement depends mainly on the epidemiological history and parotid gland and/or other acute salivary gland enlargement. Laboratory-specific examinations are required for confirmed cases.
**4. Diagnosis and Classification**	
**4.1 Suspected cases**	Any of the following is a suspected case:<br>4.1.1 Compliance with 2.2.2;<br>4.1.2: 2.1 and 2.2.1;<br>4.1.3 Agrees with 2.1 and 2.2.3;<br>4.1.4 Agrees with 2.1 and 2.2.4;<br>4.1.5 Agrees with 2.1 and 2.2.5.
**4.2 Clinically diagnosed cases**	Any of the following is a clinically diagnosed case:<br>4.2.1 Compliance with 2.2.2 and 2.2.1;<br>4.2.2 Compliance with 2.2.2 and 2.2.3;<br>4.2.3 Compliance with 2.2.2 and 2.2.4;<br>4.2.4 Compliance with 2.2.2 and 2.2.5;<br>4.2.5 Compliance with 2.1 and 2.2.1 and 2.3.1;<br>4.2.6 Compliance with 2.1 and 2.2.1 and 2.3.2;<br>4.2.7 Is consistent with 2.1 and 2.2.1 and 2.3.3.
**4.3 Confirmed cases**	In accordance with any of the following items,<br>5.3.1 Suspected cases or clinically diagnosed cases with 2.3.4<br>5.3.2 Suspected cases or clinically diagnosed cases with 2.3.5<br>5.3.3 suspected cases or clinically diagnosed cases with 2.3.6

**Chinese Standard Number**	WS 270-2007
**Issued by**	National Health Commission of the People's Republic of China
**Date issued**	April 17, 2007
**Official Source**	http://www.nhc.gov.cn/wjw/s9491/200704/38797/files/4b993dfd62834ccda16b8240ab078a00.pdf

Supplementary Table S1-5: The diagnostic criteria for rubella in China

Standard Name	Diagnostic Criteria of Rubella
**1 Scope**	This standard is applicable to the diagnosis and report of rubella and congenital rubella syndrome in all medical and health institutions and their staff at all levels in China.
**2 Terms and definitions**	RNA: ribonucleic acid<br>CRS: congenital rubella syndrome<br>IgM: immunoglobulin M<br>IgG: immunoglobulin G<br>CPE: cytopathic effect<br>RT: reverse transcription<br>PCR: polymerase chain reaction<br>ELISA: enzyme-linked immunosorbent assay
**3 Diagnostic basis**	
**3.1 Rubella**	
**3.1.1 Epidemiologic Linkage**	No rubella has ever occurred before, and a clear contact history had occurred with confirmed rubella patients within 14 days and 21 days before the onset of the disease.
**3.1.2 Clinical manifestations**	3.1.2.1 Fever, generally low or moderate<br>3.1.2.2. Red congestive maculopapular rash over the whole skin within 2 days of onset.<br>3.1.2.3 Posterior auricular, occipital, cervical lymph node enlargement or conjunctivitis or joint pain (arthritis).

**3.1.3 Laboratory Tests**	3. 1. 3.1 Rubella virus is isolated from throat swabs or urine samples, or rubella virus nucleic acid is detected.<br>3.1.3.2 Serum is positive for rubella IgM antibody (no live attenuated rubella vaccine were inoculated in the past month)<br>3. 1. 3. 3. The titres of serum rubella IgG antibody or rubella haemagglutination inhibitory antibody are more than 4 times higher in the convalescent stage than in the acute stage.<br>3.1.3.4 The antibody test is negative in the acute phase and positive in the convalescent phase.
**4. Diagnostic principles**	Clinical diagnosis is made according to clinical manifestations and epidemiology. The diagnosis is confirmed according to the results of the serum rubella antibody test or the rubella etiology test.
**5. Diagnosis and Classification**	
**5.1 Rubella**	
**5.1.1 Suspected cases**	Compliance with either of the following:<br>5.1.1.1 3.1.2.1 and 3.1.2.2<br>5.1.1.2 3.1.2.2 and 3.1.2.3
**5.1.2 Clinically diagnosed cases**	Suspected cases and concurrent coincidence 3.1.1
**5.1.3 Confirmed cases**	Simultaneous merger of suspected cases with any of 3.1.3
**Chinese Standard Number**	WS297-2008

**Issued by**	National Health Commission of the People's Republic of China
**Date issued**	December 11, 2008
**Official Source**	http://www.nhc.gov.cn/wjw/s9491/200908/42159/files/f6ed02c7a47a49f9a30570ce2adbf058.pdf

Supplementary Table S1-6: The diagnostic criteria for pertussis in China

Standard Name	Diagnostic Criteria of Pertussis
**1 Scope**	This standard is applicable to the diagnosis and reporting of pertussis by medical and health institutions and their staff at all levels in China.
**2 Terms and definitions**	
**3 Diagnostic basis**	
**3.1 Epidemiologic Linkage**	The incidence of pertussis occurs in spring and summer. Pertussis is epidemic in the area. The patient has a history of close contact with pertussis patients.<br>The patient has no history of vaccination.
**3.2 Clinical manifestations**	3.2.1 Typical cases have paroxysmal and spastic cough and persistent cough for more than 2 weeks.<br>3.2.2 Infants with atypical cases have recurrent apnea, asphyxia, cyanosis and bradycardia symptoms, or intermittent paroxysmal cough; adolescents and adults have atypical mild symptoms. Symptoms of catarrhal, spastic and convalescent stages are shortened or show no obvious stages other than a long-term cough lasting more than two weeks.
**3.3 Laboratory Tests**	3.3.1 Peripheral white blood cell count and lymphocyte count increase significantly.<br>3.3.2 Bordetella pertussis is isolated from sputum and nasopharyngeal secretions.<br>3.3.3 The serum specific antibody increases by more than 4 times in the convalescent stage than in the acute stage.
**4. Diagnostic principles**	Pertussis cases can be diagnosed based on the epidemiological history, clinical manifestations and laboratory examination results.
**5. Diagnosis and Classification**	

**5.1 Suspected cases**	Comply with any of the provisions of 3.2.1, 3.2.2, or with the provisions of 3.1
**5.2 Clinically diagnosed cases**	Suspected cases meet the requirements of 3.3.1 at the same time
**5.3 Confirmed cases**	The clinically diagnosed cases also conform to any of the provisions of 3.3.2 and 3.3.3 in laboratory tests.
**Chinese Standard Number**	WS 274-2007
**Issued by**	National Health Commission of the People's Republic of China
**Date issued**	April 17, 2007
**Official Source**	http://www.nhc.gov.cn/ewebeditor/uploadfile/2014/10/20141010173745664.PDF

Supplementary Table S1-7: The diagnostic criteria for scarlet fever in China

Standard Name	Diagnostic Criteria of Scarlet Fever
**1 Scope**	This standard applies to nationwide medical institutions and their staff to diagnose and report scarlet fever.
**2 Terms and definitions**	2.1 White strawberry tongue<br>The tongue has a white coating on it, while the papillae of the tongue are swollen and reddened, standing out on the white coating, making the tongue resemble a strawberry.<br>2.2 Red strawberry tongue<br>White strawberry tongue following the desquamating process, or the shedding of the tissue which created the white coating) the whiteness disappears while the red and enlarged papillae give it the "red strawberry" appearance.<br>2.3 Pastia's lines<br>Lines of petechiae which appear as pink/red areas located in arm pits and elbow pits.<br>2.4 Circumoral pallor<br>Obvious facial hyperemia compared to hyperemia of the nose and mouth, appearing to be white.
**3 Diagnostic basis**	
**3.1 Epidemiologic Linkage**	Local occurrence and prevalence of the disease, with exposure to scarlet fever patients or to tonsillitis, angina, otitis media, erysipelas or other streptococcal infected patients.

**3.2 Clinical manifestations**	3.2.1 Common scarlet fever<br>Abrupt onset with fever, angina, and rash. Rash is observed on the second day of fever; the skin has disseminated hyperemia and flushing, among which congestive rash as small as a needle's point can be seen that fades with pressing, accompanied by itching. A small number of patients can have a rash with yellow-white pustules, which do not break easily. Pastia's lines may form on skin creases. Facial hyperemia emerges without rash, accompanied by "circumoral pallor". In early onset of the disease, "white strawberry tongue" is seen and is more severe toward the edge of the tongue. After 2-3 days the white coating begins to fall off, forming a "red strawberry tongue". After 2-5 days the rash subsides, after which the skin has desquamation or furfur.<br>3.2.2 Mild scarlet fever<br>Fever, angina and rash are mild and short of duration; desquamation is also mild.<br>3.2.3 Toxic type<br>The main clinical manifestation is toxemia, with obvious poisoning symptoms, like fever, headache, vomiting, hemorrhagic rash, confusion, etc. Angina is not severe. Toxic myocarditis, toxic hepatitis and septic shock can occur.<br>3.2.4 Sepsis type<br>Pharyngeal swelling with exudation of pus or even ulcers, causing cervical lymphadenitis, acute otitis media, acute sinusitis, etc. Can also cause sepsis.<br>3.2.5 Surgical and obstetric type<br>Pathogenic bacteria invade from the wound or birth canal. Local rash at first, which extends to the whole body with no pharyngitis. Systemic symptoms are mostly mild.
**3.3 Laboratory Tests**	3.3.1 The total number of leukocytes and neutrophils increases, with possible toxic granulation for severe patients.<br>3.3.2 Group A *Streptococcus by* rapid antigen detection is positive.<br>3.3.3 The result of bacteria identification is the β hemolytic streptococcus by bacterial culture and by microscopy.<br>3.3.4 The result of a bacitracin-sensitive test is positive.<br>3.3.5 The result of biochemical identification is *Streptococcus pyogenes.*<br>3.3.6 Throat swab or other focal secretion is identified as Group A *β hemolytic streptococcus* by bacteria seroty

**4. Diagnostic principles**	Comprehensive diagnosis should be based on epidemiological data, clinical manifestations and laboratory tests. Confirmation must be based on etiological examination.
**5. Diagnosis and Classification**	
**5.1 Suspected cases**	Satisfying clinical manifestations in 3.2 and 3.3.1.
**5.2 Clinically diagnosed cases**	Meeting any of the following for diagnosis:<br>5.2.1 Probable cases satisfying 3.1.<br>5.2.2 Probable cases satisfying at least one of 3.3.2, 3.3.3, 3.3.4, or 3.3.5.
**5.3 Confirmed cases**	Clinical diagnosis of cases satisfying 3.3.6.
**Chinese Standard Number**	WS282-2008
**Issued by**	National Health Commission of the People’s Republic of China
**Date issued**	February 28, 2008
**Official Source**	http://www.nhc.gov.cn/wjw/s9491/200802/38805/files/2c4dd2444eb24922afca4bb3abed613c.pdf

Supplementary Table S2. Changes in the average yearly incidence rates of 8 respiratory infectious diseases in Shanghai, China

Eight Respiratory infectious diseases (RID)	Average yearly incidence (per 100,000)		Average yearly cases		Changes (%) (95%CI)	P-value
	**2020**	**2019**	**2020**	**2019**		
Influenza	93.53	185.46	13527	26823	-49.57(-51.04 to -48.10)	<.001
**Seven RID**	**2020**	**2017–2019**	**2020**	**2017–2019**	**Changes (%) (95%CI)**	**P-value**
Overall	38.10	61.26	5511	8858	-37.80(-40.45 to -35.15)	<.001
COVID-19	1.36	0.00	196	0	NA	NA
Epidemic Parotitis	6.44	10.97	931	1586	-41.30(-47.50 to -35.10)	<.001
Measles	0.03	0.19	5	27	-81.49(-122.55 to -40.42)	<.001
Pulmonary Tuberculosis	25.42	27.92	3677	4037	-8.95(-13.21 to -4.69)	<.001
Rubella	0.06	0.26	8	37	-78.38(-113.92 to -42.85)	<.001
Scarlet Fever	4.76	21.63	689	3128	-77.98(-81.85 to -74.11)	<.001
Pertussis	0.03	0.30	5	43	-88.28(-120.00 to -56.57)	<.001

Notes: Changes = $(x_2-x_1)/x_1\times100\%$, x_1: average yearly incidence in 2017-2019; x_2: average yearly incidence in 2020. The *p*-values were computed using two proportional tests.

Supplementary Table S3. Changes in the average yearly incidence rates of 8 respiratory infectious diseases in Zhejiang Province, China

Respiratory infectious diseases	Average yearly incidence (per 100,000)		Average yearly cases		Changes (%) (95%CI)	P-value
	2020	2019	2020	2019		
Influenza	537.61	949.90	270897	476788	-43.40(-43.76 to -43.05)	<.001
**Seven RID**	**2020**	**2017–2019**	**2020**	**2017–2019**	**Changes (%) (95%CI)**	**P-value**
Overall	74.05	93.02	37314	46299	-20.39(-21.61 to -19.17)	<.001
COVID-19	2.56	0.00	1291	0	NA	NA
Epidemic Parotitis	8.35	12.74	4208	6341	-34.44(-37.60 to -31.28)	<.001
Measles	0.09	0.44	46	220	-79.31(-93.83 to -64.80)	<.001
Pulmonary Tuberculosis	61.23	73.07	30853	36371	-16.21(-17.60 to -14.82)	<.001
Rubella	0.17	0.66	86	329	-74.15(-86.27 to -62.04)	<.001
Scarlet Fever	1.52	4.88	766	2429	-68.84(-73.39 to -64.30)	<.001
Pertussis	0.13	1.22	64	609	-89.62(-97.96 to -81.28)	<.001

Notes: Changes = $(x_2-x_1)/x_1\times100\%$, x_1: average yearly incidence in 2017-2019; x_2: average yearly incidence in 2020. The *p*-values were computed using two proportional tests.

Supplementary Table S4. Comparison of the average yearly incidence rates of 8 respiratory infectious diseases between Shanghai and Zhejiang, China

Respiratory infectious diseases	2017-2019 Average yearly incidence (per 100,000)					2020 Average yearly incidence (per 100,000)				
	Shanghai	Zhejiang	P-value (Difference)	Correlation coefficient	P-value	Shanghai	Zhejiang	P-value (Difference)	Correlation coefficient	P-value
Overall	143.66	495.79	<.001	0.99	<.001	131.64	611.67	<.001	1.00	<.001
COVID-19	0.00	0.00	NA	NA	NA	1.36	2.56	<.001	0.94	<.001
Epidemic Parotitis	10.97	12.74	<.001	0.91	<.001	6.44	8.35	<.001	0.85	<.001
Influenza	82.38	402.75	<.001	0.99	<.001	93.53	537.61	<.001	1.00	<.001
Measles	0.19	0.44	<.001	0.56	0.058	0.03	0.09	0.006	0.05	0.890
Pulmonary Tuberculosis	27.92	73.07	<.001	0.72	0.009	25.42	61.23	<.001	0.71	0.010
Rubella	0.26	0.66	<.001	0.96	<.001	0.06	0.17	<.001	0.03	0.929
Scarlet Fever	21.63	4.88	<.001	0.97	<.001	4.76	1.52	<.001	0.97	<.001
Pertussis	0.30	1.22	<.001	0.69	0.013	0.03	0.13	<.001	0.80	0.002

Notes: The differences in the *p*-values were computed using the two proportional tests.

Supplementary Table S5. Changes in the average monthly incidence rates of 8 respiratory infectious diseases in the emergency response stage (January to April 2020) and the routine response stage (May to December 2020) in Shanghai, China

Eight RID	Emergency stage (January to April 2020) Average monthly incidence (per 100,000)				Routine stage (May to December 2020) Average monthly incidence (per 100,000)				P value (Emergency vs. Routine)
	2020	2019	Changes (%) (95%CI)	P-value	2020	2019	Changes (%) (95%CI)	P-value	
Influenza	22.43	24.43	-8.19 (-12.75 to -3.62)	<.001	0.48	10.97	-95.65 (-100.68 to -90.62)	<.001	<.001
**Seven RID**	**2020**	**2017–2019**	**Changes (%) (95%CI)**	**P-value**	**2020**	**2017–2019**	**Changes (%) (95%CI)**	**P-value**	**P value (Emergency vs. Routine)**
Overall	2.85	4.59	-37.76(-47.46 to -28.07)	<.001	3.34	5.36	-37.81(-46.78 to -28.85)	<.001	0.994
COVID-19	0.26	0.00	NA	NA	0.04	0.00	NA	NA	NA
Epidemic Parotitis	0.26	0.69	-62.57(-85.61 to -39.54)	<.001	0.68	1.03	-34.18(-54.89 to -13.46)	0.001	0.072
Measles	0.00	0.01	-100.00(-235.79 to 35.79)	0.149	0.00	0.02	-73.22(-217.69 to 71.25)	0.321	0.791
Pulmonary Tuberculosis	1.60	2.12	-24.39(-39.23 to -9.54)	0.001	2.38	2.43	-2.23(-16.93 to 12.47)	0.766	0.038

Rubella	0.01	0.02	-78.06(-195.15 to 39.04)	0.191	0.00	0.02	-78.58(-205.04 to 47.88)	0.223	0.995
Scarlet Fever	0.73	1.72	-57.91(-72.71 to -43.12)	<.001	0.23	1.84	-87.37(-100.12 to -74.63)	<.001	0.003
Pertussis	0.01	0.02	-50.01(-201.82 to 101.79)	0.518	0.00	0.03	-100.00(-196.99 to -3.01)	0.043	0.587

Notes: Changes $=(x_2-x_1)/x_1\times100\%$, x_1: average monthly incidence in 2017-2019; x_2: average monthly incidence in 2020. The *p*-values were computed using two proportional tests. For the emergency or routine response stages, the *p*-values for the emergency versus the routine response stages were computed using two ratio tests.

Supplementary Table S6. Changes in the average monthly incidence rates of 8 respiratory infectious diseases in the emergency response stage (January to April 2020) and the routine response stage (May to December 2020) compared with the previous three years in Zhejiang Province, China

Respiratory infectious diseases	Emergency stage (January to April 2020) Average monthly incidence (per 100,000)				Routine stage (May to December 2020) Average monthly incidence (per 100,000)				P value (Emergency vs. Routine)
	2020	2019	Changes (%) (95%CI)	P-value	2020	2019	Changes (%) (95%CI)	P-value	
Influenza	131.49	113.15	16.21 (15.00 to 17.41)	<.001	1.46	62.16	-97.66 (-98.78 to -96.54)	<.001	<.001
**Respiratory infectious diseases**	**2020**	**2017–2019**	**Changes (%) (95%CI)**	**P-value**	**2020**	**2017–2019**	**Changes (%) (95%CI)**	**P-value**	**P value (Emergency vs. Routine)**
Overall	5.64	7.31	-22.76 (-27.07 to -18.44)	<.001	6.43	7.97	-19.30(-23.47 to -15.13)	<.001	0.259
COVID-19	0.63	0.00	NA	NA	0.01	0.00	NA	NA	NA
Epidemic Parotitis	0.39	0.80	-51.53(-63.44 to -39.63)	<.001	0.85	1.19	-28.67(-39.18 to -18.15)	<.001	0.005
Measles	0.00	0.04	-90.52(-135.42 to -45.61)	<.001	0.01	0.03	-72.47(-126.01 to -18.92)	0.008	0.613
Pulmonary Tuberculosis	4.43	5.99	-26.02(-30.74 to -21.30)	<.001	5.44	6.14	-11.42(-16.28 to -6.57)	<.001	<.001

Rubella	0.02	0.06	-67.07(-106.81 to -27.34)	<.001	0.01	0.05	-78.73(-121.89 to -35.57)	<.001	0.697
Scarlet Fever	0.15	0.34	-55.29(-73.45 to -37.13)	<.001	0.11	0.44	-74.03(-88.85 to -59.20)	<.001	0.117
Pertussis	0.02	0.07	-70.08(-107.97 to -32.19)	<.001	0.01	0.12	-95.38(-121.52 to -69.24)	<.001	0.281

Notes: Changes = $(x_2-x_1)/x_1\times100\%$, x_1: average monthly incidence in 2017-2019; x_2: average monthly incidence in 2020. The *p*-values were computed using two proportional tests. For the emergency or routine response stages, the *p*-values for the emergency versus the routine response stages were computed using two ratio tests.

Supplementary Table S7. Comparison of the actual and predicted average incidence rates of 8 respiratory infectious diseases in Shanghai in 2020

Respiratory infectious diseases	Average yearly incidence (per 100,000)		Average yearly cases		Changes (%) (95%CI)	P-value
	2020	2020 prediction	2020	2020 prediction		
Overall	131.63	277.69	19038	40163	-52.60(-53.78 to -51.41)	<.001
COVID-19	1.36	0.00	196	0	NA	NA
Epidemic Parotitis	6.44	11.40	931	1649	-43.55(-49.59 to -37.52)	<.001
Influenza	93.53	189.27	13527	27375	-50.59(-52.03 to -49.14)	<.001
Measles	0.03	0.19	5	27	-81.81(-122.45 to -41.17)	<.001
Pulmonary Tuberculosis	25.42	27.75	3677	4014	-8.40(-12.68 to -4.11)	<.001
Rubella	0.06	29.04	8	4200	-99.81(-102.84 to -96.78)	<.001
Scarlet Fever	4.76	19.61	689	2836	-75.71(-79.81 to -71.60)	<.001
Pertussis	0.03	0.42	5	61	-91.80(-117.91 to -65.70)	<.001

Notes: Changes $=(x_2-x_1)/x_1\times100\%$, x_1: average yearly incidence in 2020 prediction; x_2: average yearly incidence in 2020. The *p*-values were computed using two proportional tests.

Supplementary Table S8. Comparison of the true and predicted average incidence rates of 8 respiratory infectious diseases in Zhejiang Province in 2020

Respiratory infectious diseases	Average yearly incidence (per 100,000)		Average yearly cases		Changes (%) (95%CI)	P-value
	2020	2020 prediction	2020	2020 prediction		
Overall	611.67	805.49	308219	405877	-24.06(-24.46 to -23.66)	<.001
COVID-19	2.56	0.00	1291	0	NA	NA
Epidemic Parotitis	8.35	13.08	4208	6589	-36.14(-39.23 to -33.05)	<.001
Influenza	537.61	715.69	270897	360629	-24.88(-25.31 to -24.45)	<.001
Measles	0.09	0.24	46	122	-62.30(-83.12 to -41.47)	<.001
Pulmonary Tuberculosis	61.23	68.86	30853	34700	-11.09(-12.53 to -9.64)	<.001
Rubella	0.17	0.60	86	300	-71.33(-84.17 to -58.50)	<.001
Scarlet Fever	1.52	5.37	766	2708	-71.71(-75.98 to -67.44)	<.001
Pertussis	0.13	1.65	64	830	-92.29(-99.35 to -85.22)	<.001

Notes: Changes = $(x_2-x_1)/x_1\times100\%$, x_1: average yearly incidence in 2020 prediction; x_2: average yearly incidence in 2020. The *p*-values were computed using two proportional tests.

Supplementary Table S9. Comparisons of average monthly incidence of respiratory infectious diseases among 2021, 2020, and 2017–2019

Average monthly incidence in Shanghai (per 100,000)												
Diseases	2021 versus 2020				2021 versus 2019 or 2017-2019				2020 versus 2019 or 2017–2019			
	2021	2020	Changes (%)	p-value	2021	2019	Changes (%)	p-value	2020	2019	Changes (%)	p-value
Influenza	1.32	7.79	-83.12	<0.001	1.32	15.45	-91.49	<0.001	7.79	15.45	-49.57	<0.001
	2021	2020	Changes (%)	p-value	2021	2017-2019	Changes (%)	p-value	2020	2017-2019	Changes (%)	p-value
Total	3.21	3.18	1.10	0.868	3.21	5.10	-37.11	<0.001	3.18	5.10	-37.80	<0.001
COVID-19	0.05	0.11	-56.65	0.056	0.05	0	NA	NA	0.11	0	NA	NA
Epidemic Parotitis	0.59	0.54	10.80	0.512	0.59	0.91	-34.97	0.002	0.54	0.91	-41.30	<0.001
Measles	0.00	0.00	-4.27	0.984	0.00	0.02	-82.28	0.255	0.00	0.02	-81.49	0.262

**Pulmonary Tuberculosis**	2.15	2.12	1.51	0.853	2.15	2.33	-7.58	0.316	2.12	2.33	-8.95	0.235
**Rubella**	0.00	0.00	-55.13	0.708	0.00	0.02	-90.30	0.130	0.00	0.02	-78.38	0.212
**Scarlet Fever**	0.40	0.40	0.91	0.961	0.40	1.80	-77.78	<0.001	0.40	1.80	-77.98	<0.001
**Pertussis**	0.01	0.00	282.93	0.405	0.01	0.02	-55.14	0.387	0.00	0.02	-88.28	0.115
**Average monthly incidence in Zhejiang (per 100,000)**												
**Diseases**	**2021 versus 2020**				**2021 versus 2019 or 2017-2019**				**2020 versus 2019 or 2017–2019**			
	**2021**	**2020**	**Changes (%)**	**p-value**	**2021**	**2019**	**Changes (%)**	**p-value**	**2020**	**2019**	**Changes (%)**	**p-value**
**Influenza**	4.93	44.80	-89.00	<0.001	4.93	79.16	-93.77	<0.001	44.80	79.16	-43.40	<0.001
	**2021**	**2020**	**Changes (%)**	**p-value**	**2021**	**2017-2019**	**Changes (%)**	**p-value**	**2020**	**2017-2019**	**Changes (%)**	**p-value**
**Total**	5.98	6.17	-3.10	0.217	5.98	7.75	-22.86	<0.001	6.17	7.75	-20.39	<0.001
**COVID-19**	0.03	0.21	-84.29	<0.001	0.03	0	NA	NA	0.21	0	NA	NA
**Epidemic Parotitis**	0.66	0.70	-5.66	0.447	0.66	1.06	-38.15	<0.001	0.70	1.06	-34.44	<0.001

**Measles**	0.01	0.01	-29.98	0.652	0.01	0.04	-85.52	<0.001	0.01	0.04	-79.31	0.002
**Pulmonary Tuberculosis**	5.05	5.10	-1.03	0.710	5.05	6.09	-17.07	<0.001	5.10	6.09	-16.21	<0.001
**Rubella**	0.01	0.01	-37.58	0.429	0.01	0.06	-83.87	<0.001	0.01	0.06	-74.15	<0.001
**Scarlet Fever**	0.20	0.13	55.42	0.006	0.20	0.41	-51.58	<0.001	0.13	0.41	-68.84	<0.001
**Pertussis**	0.03	0.01	170.26	0.041	0.03	0.10	-71.94	<0.001	0.01	0.10	-89.62	<0.001

Notes: Changes = $(x_2-x_1)/x_1\times100\%$, x_1: average monthly incidence in 2017-2019 or 2019 or 2020; x_2: average monthly incidence in 2020 or 2021. The *p*-values were computed using two proportional tests.

Supplementary Table S10. Changes in average monthly concentration of 6 air pollutants among 2021, 2020, and 2017–2019 in Shanghai and Zhejiang Province, China

**Air pollutant**	**2021 versus 2020**				**2021 versus 2017-2019**				**2020 versus 2017–2019**			
	**2021**	**2020**	**Changes (%)**	**p-value**	**2021**	**2017-2019**	**Changes (%)**	**p-value**	**2020**	**2017-2019**	**Changes (%)**	**p-value**
**Average monthly concentration in Shanghai**												

**Overall**	200.18	214.64	-6.74	0.207	200.19	245.75	-18.54	<.001	214.64	245.75	-12.66	0.003
**SO_2 (μg/m^3)**	5.60	6.38	-12.37	0.050	5.60	9.44	-40.76	<.001	6.38	9.44	-32.40	<.001
**NO_2 (μg/m^3)**	31.10	37.02	-15.98	0.131	31.10	42.11	-26.15	0.005	37.02	42.11	-12.10	0.107
**CO (mg/m^3)**	0.59	0.66	-10.75	0.052	0.59	0.69	-14.67	<.001	0.66	0.69	-4.40	0.393
**O_3 (μg/m^3)**	95.59	97.61	-2.07	0.820	95.59	103.98	-8.07	0.279	97.61	103.98	-6.13	0.431
**$PM_{2.5}$ (μg/m^3)**	25.51	31.73	-19.61	0.137	25.51	36.39	-29.90	0.003	31.73	36.39	-12.80	0.197
**PM_{10} (μg/m^3)**	41.80	41.24	1.35	0.915	41.80	53.14	-21.34	0.043	41.24	53.14	-22.39	<.001
**Average monthly concentration in Zhejiang**												
**Overall**	193.41	198.92	-2.77	0.627	193.41	228.25	-15.26	0.001	198.92	228.25	-12.85	0.008
**SO_2 (μg/m^3)**	5.94	5.86	1.37	0.862	5.94	8.79	-32.45	<.001	5.86	8.79	-33.36	<.001
**NO_2 (μg/m^3)**	26.38	28.53	-7.55	0.560	26.38	31.92	-17.37	0.085	28.53	31.92	-10.62	0.274
**CO (mg/m^3)**	0.57	0.62	-8.07	0.224	0.57	0.74	-23.07	<.001	0.62	0.74	-16.32	0.004
**O_3 (μg/m^3)**	93.79	93.23	0.60	0.952	93.79	95.88	-2.18	0.775	93.23	95.88	-2.77	0.755
**$PM_{2.5}$ (μg/m^3)**	22.38	25.14	-11.00	0.396	22.38	34.12	-34.41	<.001	25.14	34.12	-26.30	0.004

**PM_{10} (μg/m^3)**	44.36	45.54	-2.60	0.839	44.36	56.80	-21.90	0.035	45.54	56.80	-19.82	0.015

Notes: Changes = $(x_2-x_1)/x_1 \times 100\%$, x_1: average monthly concentration in 2017-2019 or 2020, x_2: average monthly concentration in 2020 or 2021. The *p*-values were computed using t-test.
